# OTME-5. Meningioma liquid biopsy specimens exhibit contrasting immune-cell landscapes across methylation-subtypes and estimated recurrence risk subgroups

**DOI:** 10.1093/noajnl/vdab070.056

**Published:** 2021-07-05

**Authors:** Grayson Herrgott, Ruicong She, Thais Sabedot, Michael Wells, Karam Asmaro, Tathiane Malta, Maritza Mosella, Kevin Nelson, Ana deCarvalho, Laila Poisson, Abir Mukherjee, Simona Cazacu, Adam Robin, Ian Lee, James Snyder, Tobias Walbert, Mark Rosenblum, Tom Mikkelsen, Steven Kalkanis, Jack Rock, Houtan Noushmehr, Ana Valeria Castro

**Affiliations:** Henry Ford Health System, Detroit, MI, USA

## Abstract

**Background:**

Tumor-infiltrating immune cell compositions have been previously correlated to encouragement or inhibition of tumor growth. This association highlights immune-landscape profiling through non-invasive methods as a crucial step in approaches to treatment of patients with meningioma (MNG), a prevalent primary intracranial tumor. Genome-wide DNA methylation patterns can aid in definition and assessment of cell compositions in liquid biopsy serum specimens, and allow for development of machine-learning models with predictive capabilities.

**Methods:**

We profiled the cfDNA methylome (EPIC array) in liquid biopsy specimens from patients with MNG (n = 63) and nontumor controls (n = 6). We conducted both unsupervised epigenome-wide and supervised analyses of the meningioma methylome. Estimation of immune cell composition was conducted using Python-based methodology, where a reference methylome atlas of chosen cell types (B-cells, CD4- and CD8T-cells, neutrophils, natural killer cells, monocytes, cortical neuron, vascular endothelial cells, and healthy meninge) was used to deconvolute the MNG samples. Recurrence risk was estimated using an existing methylation-based Random-Forest classifier previously reported and validated, adapted to our serum-based cohort through employment of translatable meningioma subgroup-specific methylation markers (differentially methylated probes).

**Results:**

We identified four distinct genome-wide methylation subgroups (k-clusters) of MNG which presented differential tumor micro-environments across all cell types investigated. Application of the DNA methylation-based Random-Forest classifier allowed for categorization of primary MNG serum samples into estimated recurrence-risk subgroups. Significantly contrasting micro-environments for the subgroups were observed across several cell-types, with those MNG more likely to recur displaying depletion in cell types reported to improve anti-tumoral response in many tumors (e.g. T-Cells).

**Conclusions:**

DNA methylation based deconvolution allowed for detection of contrasting tumor microenvironment compositions across MNG methylation subtypes and recurrence-risk estimation subgroups. These results suggest that microenvironment profiling can be informative of probable tumor behavior and prognostic outcomes, helping guide therapeutic approaches towards treatment of patients with MNG.

